# Tolerability and Clinical Activity of Post-Transplantation Azacitidine in Patients Allografted for Acute Myeloid Leukemia Treated on the RICAZA Trial

**DOI:** 10.1016/j.bbmt.2015.09.004

**Published:** 2016-02

**Authors:** Charles Craddock, Nadira Jilani, Shamyla Siddique, Christina Yap, Josephine Khan, Sandeep Nagra, Janice Ward, Paul Ferguson, Peter Hazlewood, Richard Buka, Paresh Vyas, Oliver Goodyear, Eleni Tholouli, Charles Crawley, Nigel Russell, Jenny Byrne, Ram Malladi, John Snowden, Mike Dennis

**Affiliations:** 1 Centre for Clinical Haematology, Queen Elizabeth Hospital, Birmingham, United Kingdom; 2 Cancer Research UK Clinical Trials Unit, School of Cancer Sciences, University of Birmingham, Birmingham, United Kingdom; 3 School of Cancer Sciences, University of Birmingham, Birmingham, United Kingdom; 4 MRC Molecular Haematology Unit and Department of Haematology, Weatherall Institute of Molecular Medicine, John Radcliffe Hospital, Oxford, United Kingdom; 5 Department of Clinical Haematology, Manchester Royal Infirmary, Manchester, United Kingdom; 6 Cambridge Cancer Trials Centre, Cambridge University Hospitals NHS Foundation Trust, Cambridge, United Kingdom; 7 Centre for Clinical Haematology, Nottingham University Hospitals NHS Trust, Nottingham, United Kingdom; 8 Department of Haematology, Sheffield Teaching Hospitals NHS Trust and Department of Oncology, University of Sheffield, United Kingdom; 9 Haematology and Transplant Unit, The Christie NHS Foundation Trust, Manchester, United Kingdom

**Keywords:** Acute myeloid leukemia, Relapse, Azacitidine, Tumor antigens

## Abstract

Disease relapse is the major causes of treatment failure after allogeneic stem cell transplantation (SCT) in patients with acute myeloid leukemia (AML). As well as demonstrating significant clinical activity in AML, azacitidine (AZA) upregulates putative tumor antigens, inducing a CD8^+^ T cell response with the potential to augment a graft-versus-leukemia effect. We, therefore, studied the feasibility and clinical sequelae of the administration of AZA during the first year after transplantation in 51 patients with AML undergoing allogeneic SCT. Fourteen patients did not commence AZA either because of transplantation complications or withdrawal of consent. Thirty-seven patients commenced AZA at a median of 54 days (range, 40 to 194 days) after transplantation, which was well tolerated in the majority of patients. Thirty-one patients completed 3 or more cycles of AZA. Sixteen patients relapsed at a median time of 8 months after transplantation. No patient developed extensive chronic graft-versus-host disease. The induction of a post-transplantation CD8^+^ T cell response to 1 or more tumor-specific peptides was studied in 28 patients. Induction of a CD8^+^ T cell response was associated with a reduced risk of disease relapse (hazard ratio [HR], .30; 95% confidence interval [CI], .10 to .85; *P* = .02) and improved relapse-free survival (HR, .29; 95% CI, .10 to .83; *P* = .02) taking into account death as a competing risk. In conclusion, AZA is well tolerated after transplantation and appears to have the capacity to reduce the relapse risk in patients who demonstrate a CD8^+^ T cell response to tumor antigens. These observations require confirmation in a prospective clinical trial.

## Highlights

• Azacitidine is well tolerated in the majority of patients with acute myeloid leukemia after allogeneic stem cell transplantation.
• Administration of post-transplantation azacitidine is associated with a low risk of chronic graft-versus-host disease.
• Patients who demonstrate a CD8^+^ T cell response to tumor antigens demonstrate a lower risk of relapse.

## Graphical abstract

**Figure:**
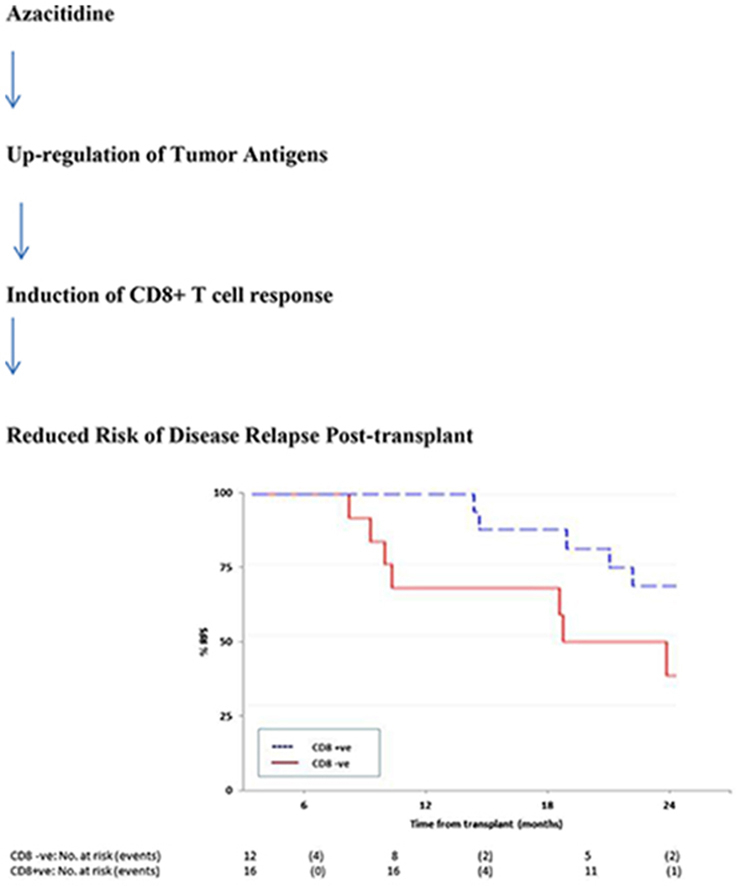

## Introduction

Allogeneic stem cell transplantation (SCT) represents an increasingly important curative option in adults with high-risk acute myeloid leukemia (AML) [1]. To a large degree, this reflects the improved tolerability of reduced-intensity conditioning (RIC) regimens, which have permitted the extension of a potentially curative graft-versus-leukemia (GVL) effect to patients up to their eighth decade 2, 3, 4. Although the increased availability of allogeneic transplantation represents a major advance in the treatment of older adults with AML, its curative potential remains limited by both disease relapse and graft-versus-host disease (GVHD), which now represent the 2 major causes of treatment failure [5]. Thirty percent to 80% of patients allografted for AML will relapse; the great majority in the first 12 months after transplantation, and the outcome for this patient population remains extremely poor [6].

The administration of cellular or pharmacological therapies with the ability to either augment a GVL effect or deliver direct antitumor activity after transplantation represents a promising strategy to reduce the risk of disease relapse. However, because the preponderance of patients allografted for AML relapse within the first year after transplantation, any intervention aimed at reducing the risk of relapse must be delivered early. Although donor lymphocyte infusions (DLI) have the potential to augment a GVL effect, they are associated with a significant risk of severe GVHD, particularly when administered early after transplantation, which complicates their routine utilization 7, 8. In contrast, pharmacological agents can, in principle, be administered early after transplantation, and consequently are the subject of increasing scrutiny as a strategy to reduce the risk of disease relapse after transplantation 9, 10, 11.

Azacitidine (AZA) is a DNA methyltransferase inhibitor that demonstrates significant clinical activity in AML and myelodysplasia 12, 13 and can also be an effective salvage therapy in patients who relapse after allogeneic transplantation 14, 15. It has been postulated that the antileukemic activity of AZA after transplantation may, in part at least, be a consequence of the upregulation of epigenetically silenced minor histocompatibility and tumor antigens on leukemic blasts, resulting in an augmented GVL response 16, 17, 18. This is supported by the observation that AZA has the capacity to induce a CD8^+^ T cell response to a range of tumor antigens in patients with AML both before and after transplantation 16, 17. Separately, AZA has been shown to augment the reconstitution of T regulatory cells (Tregs) in the immediate post-transplantation period and, in mouse models, its administration reduces the risk of severe GVHD 17, 19, 20, 21. Taken together, these data raise the possibility that post-transplantation AZA may either deliver a direct antitumor effect or epigenetically manipulate the allo-immune response, augmenting a GVL effect without increasing the risk of GVHD [22]. To date, however, there has been no systematic study of the clinical activity of adjunctive post-transplantation AZA when administered as a maintenance strategy. Two small studies have identified the maximum tolerated dose of AZA after transplantation. None to date have studied the impact of sustained administration of hypomethylating agents on clinical outcome 10, 11. We, therefore, now report clinical outcomes in a cohort of patients with AML who underwent transplantation using a standardized RIC regimen incorporating post-transplantation AZA in the RICAZA study.

## Patients and Methods

### Patient Inclusion

Adult patients with AML whose outcome with conventional chemotherapy was predicted to be poor, in whom a matched related donor or matched unrelated donor had been identified, were eligible for this trial. The clinical trial protocol was approved by the research ethics committee and all patients gave informed consent in accordance with the Declaration of Helsinki. The trial was registered at http://isrctn.org as #ISRCTN36825171. The primary endpoint of the study was to assess the safety and tolerability of AZA in patients after RIC allogeneic transplantation for AML. The impact of AZA administration on biological parameters in the first 27 patients treated on the RICAZA study have been reported in a previous publication, but with a median follow-up of 7 months, it was not possible to comment on the impact of AZA administration on clinical outcomes [17]. In this paper, we report an updated analysis of the 37 patients treated on the RICAZA study with a median follow-up of 24 months (range, 6 to 28 months).

### Transplantation Regimen

Patients who underwent allogeneic SCT using either a conditioning regimen consisting of fludarabine (30 mg/m^2^ intravenously for 5 days), melphalan (140 mg/m^2^ intravenously), and alemtuzumab (10 mg intravenously for 5 days) (n = 34) or a FLAMSA regimen (n = 3) were eligible for this trial [23]. GVHD prophylaxis consisted of cyclosporine commencing day −1 at an adjusted dose to achieve therapeutic levels between 100 μg/L and 200 μg/L after transplantation, with the aim of tapering immunosuppression in patients with no evidence of active GVHD between day 60 and day 90 after transplantation. Patients were not eligible to receive DLI in the first year after transplantation.

### AZA Schedule

Patients with stable engraftment (neutrophil count: >1 × 10^9^/L; platelet count: >50 × 10^9^/L) commenced treatment with AZA on day +42 after transplantation at a dose of 36 mg/m^2^ subcutaneously for 5 days. This was administered every 28 days up to 12 months after transplantation. Toxicity was graded against the National Cancer Institute's Common Terminology Criteria for Adverse Events. The dose of AZA was reduced to 24 mg/m^2^ in patients experiencing grade 3 or 4 hematologic toxicity that persisted for >2 weeks. Initial data concerning the tolerability of post-transplantation AZA in a cohort of 27 patients, of whom only 17 had received more than 6 courses of post-transplantation AZA, were presented in a previous publication [17] and we now report the final toxicity and clinical outcome data in the complete trial cohort of 37 patients. The presence of active acute GVHD or a history of GVHD were not exclusion criteria to trial entry. Clinical responses and transplantation outcome were assessed every 3 months by sequential bone marrow aspirates and peripheral blood lineage specific chimerism analyses, as previously described, until 12 months after transplantation.

### Quantification of Circulating Tumor-specific CD8^+^ T Cells

The number of circulating tumor-specific cytotoxic T lymphocytes was measured in peripheral blood mononuclear cells prepared from 50 mL of fresh peripheral blood using a CD137 expression and enrichment assay (Miltenyi Biotec, Bergisch Gladbach, Germany). The procedure was conducted as previously described with some minor adjustments 17, 24, 25. The HLA type of each patient was known and peptides that matched the HLA type were chosen from the list of previously described tumor-associated antigens [17]. The frequency of CD137^+^ antigen-specific T cells was calculated as a percentage of the total CD8^+^ T cell pool from the pre-enrichment estimates. The postenrichment analysis was used to validate the results obtained in pre-enrichment samples.

### Statistical Analysis

Safety and tolerability outcomes were defined in terms of hematological and nonhematological toxicities. Studied outcomes included rates of complete remission (CR), relapse-free survival (RFS), and overall survival (OS). *RFS* was defined as the time from transplantation to relapse or death, censoring alive patients at date last seen. *OS* was defined as time from transplantation to death, censoring alive patients at date last seen. The sample size was calculated using A'Herns single stage design and was based on the primary outcome measure of tolerability. A tolerability rate of 50% or less was deemed to be unacceptable and the probability of obtaining a false positive result was set at 5%. A tolerability rate of 70% was deemed to be an acceptable figure and the probability of a false negative result (ie, incorrectly rejecting for further study a treatment with a true tolerability rate of >70%) was set at 10%. The analysis reported is based on the per-protocol population, including all patients who received the protocol-defined RIC regimen and commenced AZA after transplantation. Statistical analyses were performed using STATA 12 and R version 3.1.

## Results

### Patient Demographics

Fifty-one patients were registered for treatment on the RICAZA trial and underwent allogeneic transplantation. Fourteen patients did not commence AZA therapy because of post-transplantation complications, including infection (n = 8), patient withdrawal of consent or ineligibility (n = 5), or acute GVHD (n = 1). Thirty-seven patients commenced monthly courses of AZA at a median time of 54 days after transplantation (range, 40 to 194 days) and are the subject of this report. The median follow-up for alive patients was 24 months (range, 6 to 28 months). The median age of the 37 patients who commenced AZA was 60 years (range, 40 to 71 years) (Table 1). Twenty-four patients (65%) were in CR1, 8 patients (22%) were in CR2, 3 patients (8%) were in first relapse, and 2 patients (5%) had primary refractory disease (Table 1). Thirteen (35%) patients underwent transplantation using a matched related donor and 24 (65%) had an adult volunteer unrelated donor. Thirty-four patients received granulocyte colony–stimulating factor–mobilized peripheral blood stem cells and 3 had bone marrow as the stem cell source. All patients engrafted with a median time to neutrophil engraftment of 13 days (range, 1 to 22 days) and a median time to platelet engraftment of 13 days (range, 10 to 33 days).

### Tolerability of Post-transplantation AZA

AZA was well tolerated in the majority of patients. Hematological and nonhematological toxicities experienced by 10% or more of patients are described in Table 2. Four patients experienced treatment delays due to neutropenia or thrombocytopenia. The most common nonhematological toxicities observed were abnormalities of liver function, injection site reaction, nausea, and infection. Thirty-one patients completed at least 3 cycles of AZA and 16 patients completed 10 cycles. Twenty patients discontinued AZA before 12 months after transplantation because of disease relapse (n = 10), infection or hematological toxicity (n = 6), or miscellaneous reasons (eg, physician decision to administer DLI, withdrawal of consent, and protocol deviation) (n = 4).

### Chimerism, GVHD, Relapse, and Outcome

At day +90 after transplantation, 22 (59%) patients demonstrated full donor chimerism in whole blood, of whom 7 (19%) demonstrated full donor chimerism in the T cell fraction. Serial chimerism studies are available on 14 patients who received AZA after transplantation, which demonstrate broad stability of T cell chimerism with no significant changes observed over time. Grade 1 or 2 acute GVHD was documented in 17 patients. No patient developed severe (grades 3 or 4) acute GVHD during the period of treatment on the RICAZA trial, although 1 patient developed severe GVHD after being withdrawn and receiving DLI for the treatment of disease relapse. Ten patients developed limited chronic GVHD but no patient developed extensive chronic GVHD.

The day 100 nonrelapse mortality was 0% and the 1-year nonrelapse mortality was 8% (all infectious deaths). A total of 19 (51%) patients died (disease relapse, n = 16; infection, n = 2; DLI-induced GVHD, n = 1). The median time to disease relapse was 8 months after transplantation (range, 5 to 11 months). The 1-year and 2-year OS were 81% (95% confidence interval [CI], 69% to 95%) and 49% (95% CI, 35% to 68%), respectively. The 1-year and 2-year RFS were 57% (95% CI, 43% to 75%) and 49% (95% CI, 35% to 68%), respectively.

### Correlation of Peripheral Blood Antitumor CD8-specific T Cell Response with Clinical Outcome

**Figure 1:**
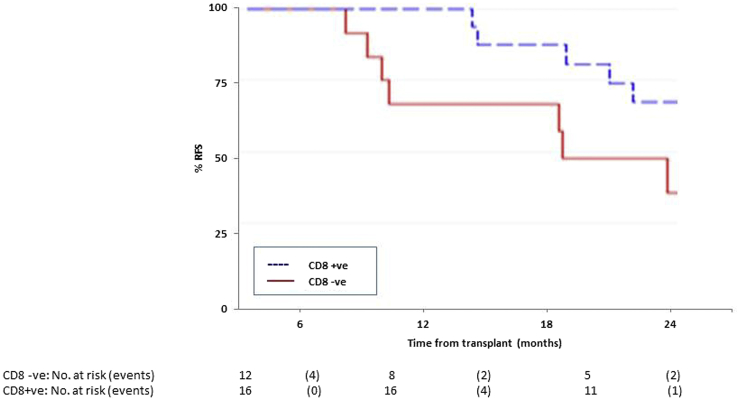
RFS of patients according to post-transplantation CD8^+^ T cell response to tumor antigens.

Twenty-eight patients treated with AZA were screened for antitumor CD8^+^ T cell responses to a range of tumor antigens. AZA induced a CD8^+^ T cell response to 1 or more tumor-specific peptides in 16 patients. A summary of their characteristics by CD8 T cell response is displayed in Table 3. As previously reported, no CD8^+^ T cell response was detected in 7 patients allografted using a similar conditioning regimen who did not receive AZA after transplantation [17]. The frequency of CD8^+^ T cells directed against the studied tumor antigens ranged from .01% to 1.6% (mean, .45%) of circulating CD8^+^ T cells, which is comparable to the frequency of cytomegalovirus-specific T cell responses detectable in cytomegalovirus-seropositive patients. Induction of a CD8^+^ anti-tumor response was associated with a reduction in relapse risk taking into account death as a competing risk (hazard ratio [HR], .30; 95% CI, .10 to .85; *P* = .02) and improved RFS (HR, .29; 95% CI, .10 to .83; *P* = .02) (Figure 1). The beneficial impact of detection of a CD8^+^ T cell response to candidate tumor antigens on relapse risk and RFS is still observed, with adjustment for time to start of AZA therapy (HR, .30; 95% CI, .1 to .94; *P* = .039) or if restricted to only 24 patients who commenced AZA before day +90 after transplantation (HR, .32; 95% CI, .10 to .95; *P* = .041) in the competing risk analysis.

The impact of detection of a CD8^+^ T cell response on relapse risk remains strong with adjustment of donor type (HR, .26; 95% CI, .09 to .70; *P* = .008) and with additional adjustment of start time to AZA therapy after transplantation (HR, .27; 95% CI, .09 to .82; *P* = .022). There was no correlation between the timing or magnitude of the CD8^+^ tumor response and relapse risk.

## Discussion

The ability of epigenetic therapies to upregulate tumor antigens represents a novel strategy by which a GVL response may be augmented. In this small prospective trial, we observed a reduced risk of disease relapse in patients treated with post-transplantation AZA who developed a CD8^+^ T cell response to a range of candidate tumor antigens. It is important to emphasize the importance of repeating this observation in a larger randomized study, but these data are supportive of further investigation of the impact of other post-transplantation epigenetic therapies, such as DNA methyltransferase inhibitor and histone deacetylase inhibitors, on transplantation outcome. Of interest, the dose of AZA observed to induce a CD8^+^ T cell response in this study is approximately one-half that utilized in the treatment of patients with *de novo* AML or myelodysplasia, consistent with the hypothesis that the observed reduction in relapse is consequent upon manipulation of the alloreactive response. Our study appears to refute the possibility that AZA might improve/outcome by simply postponing relapse, although a larger study will clearly be required to conclusively address this possibility. Because the great majority of patients destined to relapse after an allogeneic transplantation for AML will do so within the first year, a potential advantage of AZA administration, as opposed to DLI, is the ability to commence treatment early [6]. The majority of patients in this study were not only able to commence AZA within 3 months after transplantation but also complete the scheduled course of 1-year treatment. Nonetheless, approximately one third of the patients registered to this study did not receive AZA, emphasizing the potential limitations of post-transplantation interventions as a strategy to reduce the risk of disease relapse. Alternative approaches to selectively augment a GVL effect include vaccination to tumor antigens, such as WT1, and it would be of interest to combine such a strategy with AZA, as has been done for DLI 26, 27. Furthermore, although in this study we measured T cell responses to a broad range of candidate tumor antigens, future studies should be aimed at correlating clinical outcome with the induction of immune responses to specific tumor antigens.

A number of studies have demonstrated the ability of AZA to accelerate the reconstitution of Tregs after allogeneic SCT 17, 21, and in murine models this has been correlated with a reduction in the incidence of GVHD [19]. Although the observation that no patient who received post-transplantation AZA had extensive chronic GVHD is striking, particularly given the fact that most patients underwent transplantation using an unrelated donor, it is important to note that the RIC regimen utilized included alemtuzumab, which is known to reduce the incidence of both acute and chronic GVHD 28, 29. Nonetheless, these data support further examination of the impact of post-transplantation AZA on the incidence of acute or chronic GVHD. Reassuringly, our data suggest that the observed induction of Tregs and attendant absence of extensive chronic GVHD do not appear to be associated with an increased risk of disease relapse [22].

This is the first study to examine the impact of the post-transplantation administration of AZA on transplantation outcome. The reduced relapse rate in patients who demonstrate a CD8^+^ T cell response directed against tumor antigens, coupled with the absence of chronic extensive GVHD, is consistent with the hypothesis that AZA has the capacity to epigenetically manipulate the alloreactive effect after transplantation. These observations require confirmation in a prospective randomized trial.

## Figures and Tables

**Table 1:** Demographics of Study Population

Characteristic	Value
Diagnosis	
AML, de novo	24
AML, secondary	13
Karyotype	
Intermediate	30
Poor	7
Age, median (range), yr	60 (40-71)
Sex	
Male	21
Female	16
Disease status at time of transplantation	
CR1	24
CR2	8
First relapse	3
Primary refractory disease	2
Conditioning treatment	
Fludarabine, melphalan, alemtuzumab	34
Fludarabine, cytarabine, amsacrine	3
Donor type	
Sibling	13
Matched unrelated donor	24
CMV status (patient/donor)	
Positive/positive	14
Positive/negative	6
Negative/positive	3
Negative/negative	14
Stem cell source	
Peripheral blood	34
Bone marrow	3

CMV indicates cytomegalovirus.

**Table 2:** Summary of Hematological and Nonhematological Adverse Events Occurring in >10% of the Patient Population

	Grades 1-2	Grades 3-4	Total
Hematological Adverse Event			
Anemia	16	10	26
Thrombocytopenia	10	13	23
Neutropenia	3	10	13
Nonhematological Adverse Event			
Laboratory investigations – biochemistry	75	10	85
Gastrointestinal (inc. nausea, vomiting, diarrhea, constipation, anorexia)	71	2	73
Infection	35	19	54
Injection site reaction	28	0	28
Pain	21	2	23
Dermatology/skin (rash, mucositis, pruritus, shingles, dry skin, bruising, itching, peeling epidermis, skin breakdown)	21	0	21
Fatigue/lethargy	20	0	20
Pulmonary/upper respiratory (cough, dyspnea, hypoxia)	12	1	13
Neurology (headache, depression, apnea, syncope)	7	2	9
Fever	6	1	8
Cold/flu-like symptoms	8	0	8
Edema	4	0	4

**Table 3:** Demographics of Patients with CD8 T Cell Response

Characteristic	CD8 Negative (n = 12)	CD8 Positive (n = 16)
Age, years (mean, range)	61 (41-70)	59 (49-71)
Sex		
Male	7	10
Female	5	6
Disease status at time of transplantation		
CR1	8	11
CR2	3	4
First relapse	1	1
Donor type		
Sibling	4	8
Matched unrelated donor	8	8
CMV status (patient/donor)		
Positive/positive	4	7
Positive/negative	3	2
Negative/positive	0	3
Negative/negative	5	4
Stem cell source		
Peripheral blood	11	15
Bone marrow	1	1
Time from transplantation to start of AZA, median (range), d	47 (40-81)	57 (40-194)
